# Smurf1 Facilitates Oxidative Stress and Fibrosis of Ligamentum Flavum by Promoting Nrf2 Ubiquitination and Degradation

**DOI:** 10.1155/2023/1164147

**Published:** 2023-04-08

**Authors:** Yifei Gu, Jinquan Hu, Chen Wang, Min Qi, Yu Chen, Wenchao Yu, Zhanchao Wang, Xinwei Wang, Wen Yuan

**Affiliations:** Department of Orthopedics, Shanghai Changzheng Hospital, Naval Medical University, Shanghai 200003, China

## Abstract

Lumbar spinal stenosis (LSS), which can lead to irreversible neurologic damage and functional disability, is characterized by hypertrophy and fibrosis in the ligamentum flavum (LF). However, the underlying mechanism is still unclear. In the current study, the effect of Smurf1, a kind of E3 ubiquitin ligase, in promoting the fibrosis and oxidative stress of LF was investigated, and its underlying mechanism was explored. The expression of oxidative stress and fibrosis-related markers was assessed in the tissue of lumbar spinal stenosis (LSS) and lumbar disc herniation (LDH). Next, the expression of the top 10 E3 ubiquitin ligases, obtained from Gene Expression Omnibus (GEO) dataset GSE113212, was assessed in LDH and LSS, and confirmed that Smurf1 expression was markedly upregulated in the LSS group. Furthermore, Smurf1 overexpression promotes the fibrosis and oxidative stress of LF cells. Subsequently, NRF2, an important transcription factor for oxidative stress and fibrosis, was predicted to be a target of Smurf1. Mechanistically, Smurf1 directly interacts with Nrf2 and accelerates Nrf2 ubiquitination and degradation. In conclusion, the current study suggests that Smurf1 facilitated the fibrosis and oxidative stress of LF and induced the development of LSS by promoting Nrf2 ubiquitination and degradation.

## 1. Introduction

Lumbar spinal stenosis (LSS) is one of the most common spinal disorders in aging patients and is closed related to lower back pain, limb numbness, and intermittent claudication [1, 2]. Compression of the cauda equina and lumbar nerve roots often results in sensory and motor dysfunction of the lower limbs, which can lead to severe disability [3]. LSS development has been attributed to a number of factors, including disc protrusion, facet joint degeneration, and hypertrophy of the ligamentum flavum (HLF) [4, 5]. In previous studies, fibrosis was considered to be the main pathology of HLF. Histologically, normal LF consists of approximately 20% collagen fibers and 80% elastic fibers [4, 6]. Conversely, HLF tissues exhibit fibrosis changes with an increase in collagen fibers and elastic fiber loss. Recently, several studies have reported the fibrosis of HLF at the cellular and histological levels. However, to date, the molecular mechanisms underlying the fibrosis of HLF are still unclear.

Reactive oxygen species (ROS) generation leads to oxidative stress when it surpasses the capacity of antioxidant enzymes, and excessive ROS production has been linked to a number of aging illnesses. [7, 8]. Strong inflammatory reactions and fibrosis of vital organs, including the heart, kidneys, lungs, and liver, can be induced by an excess of ROS. A previous study clarified that catalase expression was decreased in HLF tissue of LSS patients [9]. Another study reported that oxidative stress mediates age-related HLF by promoting fibrosis, inflammation, and apoptosis via promoting MAPK-AKT pathway [10]. Together, these studies confirmed that oxidative stress-mediated LF fibrosis plays an important role in the development of LSS, however the exact mechanism by which oxidative stress dysregulation in HLF occurs is still unclear.

Previous research has reported that ubiquitination contributes to the age-related diseases. Hunt et al. reported that antagonistic control of myofiber size and muscle protein quality is controlled by the ubiquitin ligase UBR4 during aging [11]. Kong et al. reported that the prostaglandin D2/DP1 axis suppresses age-related Th1 activation and subsequent hypertensive response in male mice through an increase in NEDD4L-mediated T-bet degradation by ubiquitination [12]. Moreover, multiple lines of evidence indicate an important function of ubiquitination in regulating oxidative stress and fibrosis. Wang et al. reported that FBW7 regulates pulmonary epithelial stem cell senescence and fibrosis by regulating telomere uncapping [13]. However, the regulatory mechanisms of ubiquitination in the development of HLF are still unclear.

Nuclear factor-erythroid 2-related factor 2 (Nrf2) is an important regulator of many antioxidant enzymes [14]. Nrf2 regulates the balance of cell redox by facilitating the activity of antioxidant defense components, including heme oxygenase-1 (HO-1), superoxide dismutase (SOD), peroxidase (GSH-Px), and glutathione [15], and dysregulation of Nrf2 is associated with a variety of oxidative stress-related diseases, including neurodegenerative diseases [16], cardiovascular disorders [17], pulmonary diseases [18], and cancer [19]. In addition, Nrf2 activation prevents cell senescence, whereas inhibiting the activation of Nrf2 markedly accelerates cell senescence [20], suggesting that has an anti-aging impact. Additionally, Nrf2 expression and activity decreased with age. In the current study, the expression of Smurf1, an E3 ubiquitin ligase was found to be obviously increased in the LSS group, and Smurf1 overexpression accelerates oxidative stress and fibrosis of LF cells. Moreover, Smurf1 facilitates ubiquitination-mediated degradation of Nrf2.

## 2. Materials and Methods

### 2.1. Patients and Sample Harvest

All experimental protocols were approved by the Ethics Committee of the Naval Medical University (2016SL-034-01). Ligamentum flavum samples were collected from 27 patients (10 females and 17 males) who underwent posterior lumbar decompression surgery with removal of LF tissue from June 2021 to December 2021 (Table 1). For the HLF group, 15 LF specimens were harvested from LSS patients with LF hypertrophy, and for the control group, 12 specimens were collected from individuals with uncomplicated lumbar disc herniation and no LF hypertrophy. The thickness of the LF was quantified at the facet joint level on T2-weighted magnetic resonance imaging (MRI) for all 27 patients using Picture Archiving and Communication Systems (PACS) software. An expert spine surgeon assessed the value three times for each patient, and the average value designated the LF thickness. According to previous studies, hypertrophy of LF was defined as LF thickness> 4 mm [21, 22]. Extensive or partial laminectomy with LF resection was performed in all patients during the operation. The resected ligamentum flavum was rinsed in 4°C physiological saline and then sent for examination immediately.

### 2.2. Bioinformatic Analysis

The gene expression profile of data GSE113212 was obtained from the Gene Expression Omnibus (GEO) database (http://www.ncbi.nlm.nih.gov/geo/), National Center for Biotechnology Information (NCBI). This data set was based on the GPL17077 platform and contained a total of 8 samples, including 4 hypertrophic ligamentum flavum samples from the elderly individuals and 4 non-hypertrophic samples from the young individuals. The top 10 differentially expressed E3 ubiquitin ligase were obtained from this dataset to investigate the regulation of ubiquitination in the development of HLF. UbiBrowser (http://ubibrowser.ncpsb.org.cn/ubibrowser/), a target prediction tool was applied to predict the target of selected ubiquitin ligase.

### 2.3. Quantitative Real-Time PCR (qPCR)

About three cubic metres of ligamentum flavum tissue were homogenized in 800 *μ*L of Trizol. Total RNA was isolated by Trizol reagent (Invitrogen, USA) according to the manufacturer's instructions, and further reverse transcribed by using the iScript cDNA Synthesis kit (bio-rad). Real-time PCR and analysis were performed as previously described [23]. The fold changes of target genes were analyzed by the 2-*ΔΔ*Ct method and 18 s was used as an internal control.

### 2.4. Western Blotting Analysis

Western blotting was carried out as previous [23]. In brief, the total protein of tissue from LSS and LDH patients was extracted by using a commercial kit (BC3701, Solarbio, China), The following primary antibodies were used: anti-Collagen III (1 : 500, Abcam, ab6301); anti-Collagen I (1 : 1000, Abcam, ab138492); anti-a-SMA (0.5 *μ*g/ml, Abcam, ab7817); anti-Smurf1 (1 : 1000, Abcam, ab57573); anti-Nrf2 (1 : 1000, Abcam, ab62352); anti-ubiquitin (1 : 1000, Abcam, ab140601); anti-GAPDH (1 : 5000, Abcam, ab9485).

### 2.5. Immunohistochemistry (IHC)

For IHC, LSS tissue that had been formalin-fixed and paraffin-embedded was divided into 5 m serial slices. IHC was performed as previously. Primary antibodies: anti-Collagen I (1 : 1500, Abcam, ab138492); anti-Collagen III (1 : 200, Abcam, ab6301); anti-a-SMA (0.05 *μ*g/ml, Abcam, ab7817); anti-Smurf1 (1 : 1000, Abcam, ab57573).

### 2.6. Human LF Cell Isolation

Ligamentum flavum cells were isolated as described previously [21, 24]. In brief, LF tissue was washed by PBS 3 times, cut into small pieces measuring around 0.5 mm^3^ and digested for one hour with 0.2% type I collagenase (Gibco), The digested fragments were then rinsed in DMEM (Gibco), supplemented with 10% FBS (Glpbio, USA), and 100 U/ml penicillin. Cells after the third passage were used for experiments.

### 2.7. Reactive Oxygen Species (ROS) Assay

The level of ROS in LF tissue was assessed by a Tissue Reactive Oxygen Species (ROS) Detection Kit (Bestbio China) according to the instructions. The level of ROS in LF cells was assessed by a C11-BODIPY probe assay kit (Invitrogen) according to the instructions. 1 × 104 LF cells were seeded in 96-well plates and cultured for 30 minutes with 2 *μ*M C11-BODIPY probe, and the amount of ROS was measured using a flow cytometer.

### 2.8. MDA and GSH Content

The MDA and GSH content in tissue homogenates and cell lysis were analyzed by a lipid peroxidation kit (Sigma, MAK085) and Glutathione Assay Kit (Sigma, CS0260) in accordance with the standard protocol.

### 2.9. Transfection

To overexpress Smurf1, lentivirus production of Smurf1 was purchased from GeneChem (Shanghai, China) and infected LF cells according to the instructions. After 24 hours of transfection, cells were grown for additional 24 hours after transfection and then extracted for the following experiment.

### 2.10. Co-Immunoprecipitation (co-IP)

After being lysed in NP-40 lysis solution, LF cells lysate were added to the immunoprecipitation complex and rotated overnight at 4°C after being coated with anti-Smurf1 or anti-Nrf2 antibodies for 4 hours. The following day, PBS was used to rinse the Protein A/G beads three times. The immunoprecipitates complex was then examined using anti-Smurf1 or anti-NRF2 antibodies in western blotting.

### 2.11. Statistical Analysis

Data from every experiment are presented as mean ± SD. The statistical analyses were performed by SPSS 20.0. The student's *t*-test was used to assess the significance between two groups, and more than two groups was determined by one-way ANOVA followed by Tukey-Kramer multiple comparisons test was applied to determine the data for more than two groups. A statistically significant difference was defined as *p* < 0.05.

## 3. Results

### 3.1. Fibrosis and Oxidative Stress Were Upregulated in LF Tissues from HLF Patients

**Figure 1:**
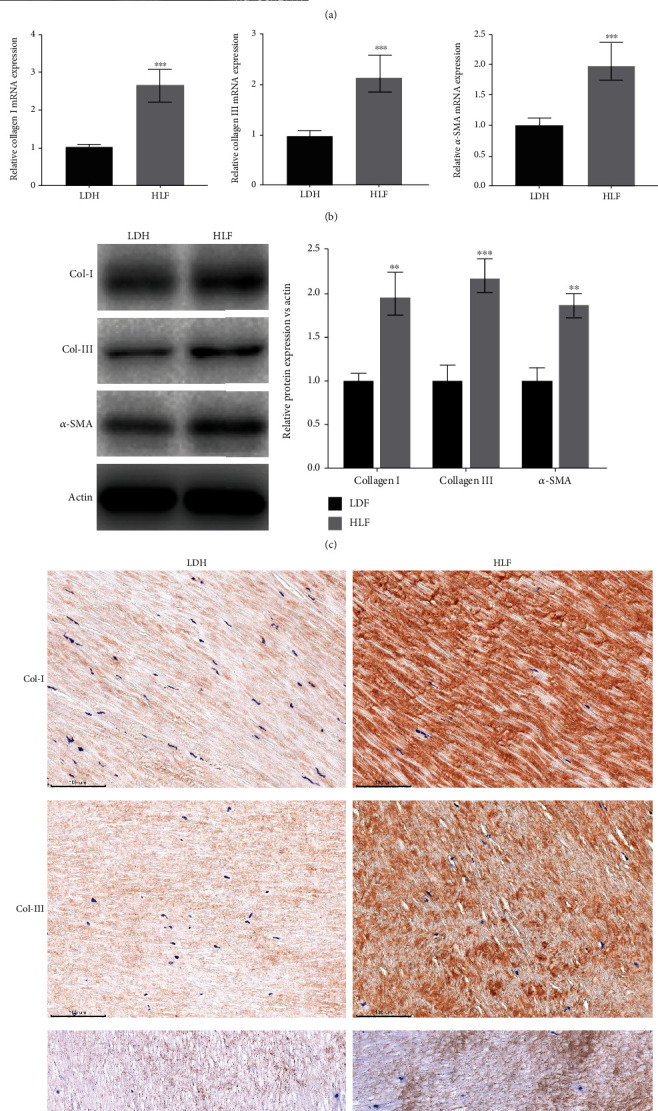
Fibrosis was upregulated in LF tissues from HLF patients. (a) Magnetic resonance imaging (MRI) shows axial views of the lumbar spinal canal in the LDH and HLF patients. (b) Collagen I, Collagen III, and *α*-SMA mRNA levels in the LDH and HLF patients were assessed by qRT-PCR (*n* = 27). ^∗∗∗^*p* < 0.001. (c, d) Collagen I, Collagen III, and *α*-SMA protein expression in the LDH and HLF patients was assessed by western-blot and IHC (*n* = 10). ^∗∗^*p* < 0.01, ^∗∗∗^*p* < 0.001.

**Figure 2:**
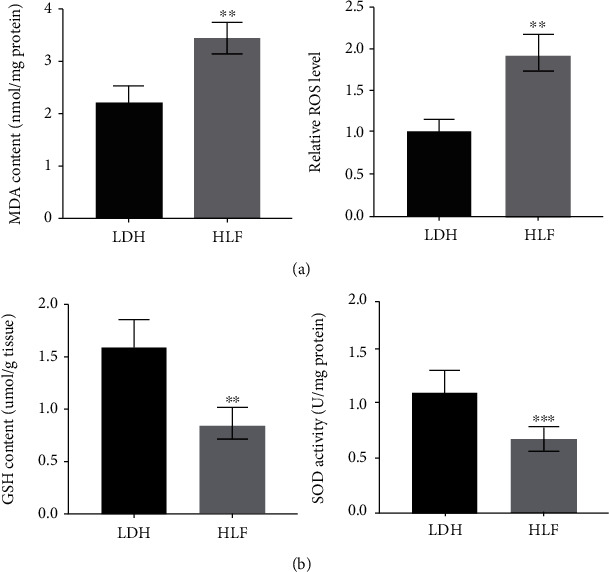
Oxidative stress was upregulated in LF tissues from HLF patients. (a) The ROS levels and malondialdehyde (MDA) were higher in the HLF group compared with the LDH group. (*n* = 27). ^∗∗^*p* < 0.01. (b) The glutathione (GSH) content, and superoxide dismutase (SOD) activity were decreased in the HLF group compared with the LDH group (*n* = 27). ^∗∗^*p* < 0.01, ^∗∗∗^*p* < 0.001.

To clarify that the HLF is the major cause of LSS, the thickness of ligamentum flavum was assessed via MRI.  Figure 1(a) showed that the thickness of LF was markedly increased in HLF patients. Meanwhile, previous studies have confirmed that the importance of LF fibrosis in the pathological progression of LSS. Thus, the expression of Collagen I, Collagen III, and *α*-SMA was assessed by qRT-PCR and western blotting in the LF tissues of HLF and LDH. We found that the expression of Collagen I, Collagen III, and *α*-SMA was markedly increased in the HLF group (Figures 1(b) and 1(c)). Moreover, the result was further confirmed by IHC staining (Figure 1(d)). Given that oxidative stress regulates age-related HLF by promoting fibrosis, oxidative stress markers expression in the HLF group were determined by ELISA. Figures 2(a) and 2(b) showed that the MDA content and ROS level were significantly increased in the HLF group, whereas the GSH content and SOD activity were markedly decreased in the HLF group. These data demonstrated that oxidative stress and ligamentum flavum fibrosis were significantly increased in HLF.

### 3.2. Smurf1 Was Upregulated in LF Tissues from HLF Patients

**Figure 3:**
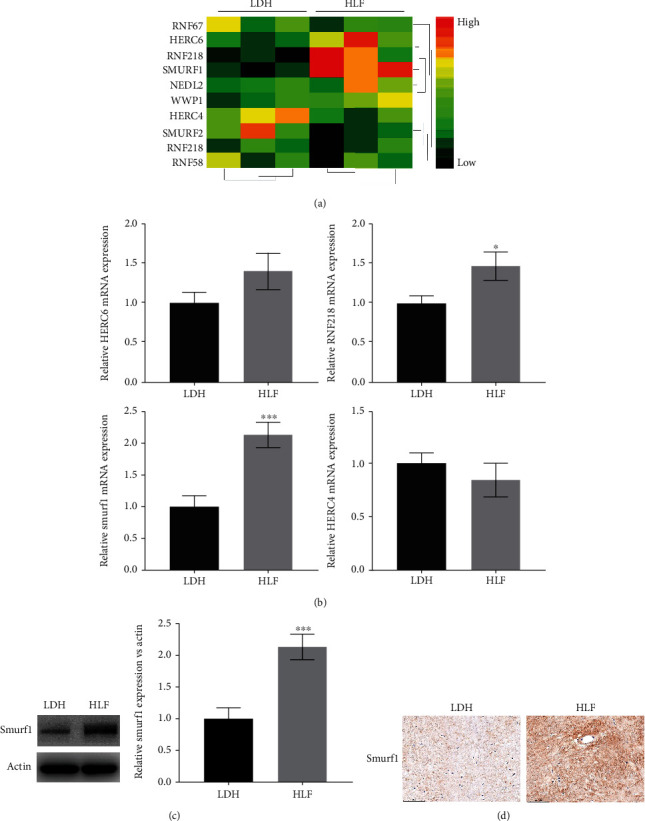
Smurf1 was upregulated in LF tissues from HLF patients. (a, b) The mRNA expression of the top ten E3 ligases was assessed by qRT-PCR array in the HLF and LDH group (*n* = 27). ^∗^*p* < 0.05. ^∗∗∗^*p* < 0.001. (c) Western blotting and semiquantification for Smurf1 expression in the HLF and LDH groups (*n* = 10). ^∗∗∗^*p* < 0.001. (d) Immunohistochemistry for Smurf1 expression in the HLF and LDH groups.

To investigate the regulation of ubiquitination in the development of LSS, the top 10 (RNF67, HERC6, RNF218, SMURF1, NEDL2, WWP1, HERC4, SMURF2, RNF218, and RNF58) differentially expressed E3 ubiquitin ligase were obtained from GSE113212, and the expression of these 10 genes was assessed in HLF and LDH patients using qRT-PCR. Figures 3(a) and 3(b) showed that the mRNA level of RNF218 and Smurf1 was markedly increased in HLF patients. Given that the Smurf1 mRNA expression was most upregulated in the HLF group, the Smurf1 protein expression was further confirmed by western blotting and IHC. Similar to the qRT-PCR results, western blotting and IHC data showed that the expressions of Smurf1 were significantly increased in HLF patients compared with LDH patients (Figures 3(c) and 3(d)), suggesting that Smurf1 may contribute to the development of HLF.

### 3.3. Smurf1 Facilitated the Fibrosis and Oxidative Stress of LF Cells

**Figure 4:**
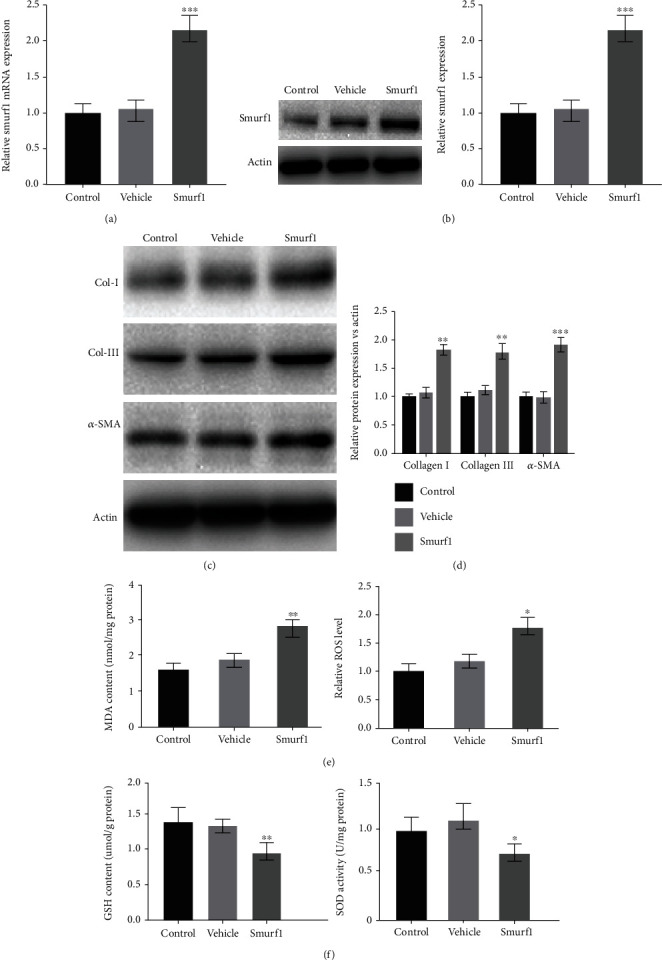
Smurf1 facilitated the fibrosis and oxidative stress of LF cells. (a, b) the transfection efficiency of Smurf1 was analyzed by qRT-PCR and western-blot. ^∗∗∗^*p* < 0.001. (c, d) Western blotting and semiquantification for Collagen I, Collagen III, and *α*-SMA mRNA levels in the LF cells with or without Smuf1-OE). ^∗∗^*p* < 0.01. ^∗∗∗^*p* < 0.001. (e) The ROS levels and MDA content was assessed in the LF cells with or without Smuf1-OE. ^∗^*p* < 0.05. ^∗∗^*p* < 0.01. (f) the Glutathione (GSH) content, and Superoxide dismutase (SOD) activity was assessed in the LF cells with or without Smuf1-OE. ^∗^*p* < 0.05. ^∗∗^*p* < 0.01.

To investigate whether Smurf1 is critical for the fibrosis and oxidative stress of HLF, Smurf1 was forcefully expressed by pcDNA-Smurf1, and the efficiency was determined by qRT-PCR and western blotting (Figures 4(a) and 4(b)). We next verify the function of Smurf1 on fibrosis and oxidative stress of LF cells. As expected, Collagen I, Collagen III, and *α*-SMA expression was significantly increased by Smurf1 (Figures 4(c) and 4(d)), suggesting that Smurf1 promoted the fibrosis of LF cells. Similarly, the oxidative stress of LF cells was upregulated by Smurf1 as evidenced by the upregulation of MDA content and ROS level (Figure 4(e)) and the downregulation of GSH content and SOD (Figure 4(f)). In conclusion, these data indicated that Smurf1 facilitated the fibrosis and oxidative stress of LF cells.

### 3.4. Smurf1 Facilitated the Fibrosis and Oxidative Stress of LF Cells by Promoting the Ubiquitination and Degradation of Nrf2

**Figure 5:**
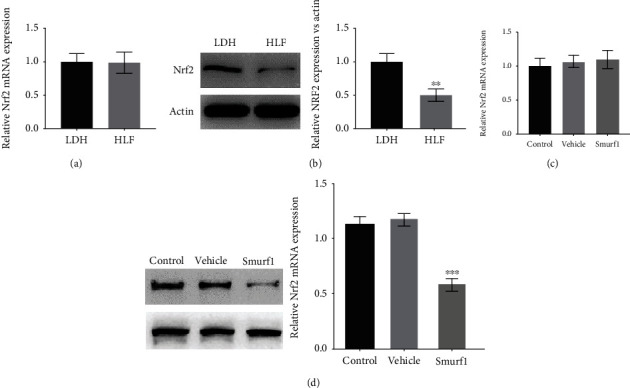
Nrf2 is the target of Smurf1. (a) Nrf2 mRNA level in the LDH and HLF patients was assessed by qRT-PCR (*n* = 27). (b) Western blotting and semiquantification for Nrf2 level in the LDH and HLF patients (*n* = 10). ^∗∗^*p* < 0.01. (c) qRT-PCR was used to determine the level of Nrf2 mRNA in LF cells with or without Smuf1-OE. (d) Western blotting and semiquantification for Collagen I, Collagen III, and *α*-SMA mRNA level in the LF cells with or without Smuf1-OE. ^∗∗∗^*p* < 0.001.

Previous studies found that Smurf1 promotes oxidative stress and fibrosis in the kidney by regulating the polyubiquitination of Nrf2 [25]. Given that Nrf2 is an important transcriptional inhibitor of oxidative stress and fibrosis, we speculated that Smurf1 might facilitate the fibrosis and oxidative stress of LF cells by regulating Nrf2. To this end, UbiBrowser was applied to predict the target of Smurf1 and Nrf2 (gene name: NFE2L2) was found to be a potential target of Smurf1 (Supplementary Figure 1). Next, the expression of Nrf2 was analyzed in LF tissues of HLF and LDH by qRT-PCR and western blotting. As shown in Figures 5(a) and 5(b), there was no significant difference in the mRNA level of Nrf2 between the LDH group and the HLF group, whereas the protein level of Nrf2 was significantly increased in the HLF group compared with the LDH group. Similarly, Smurf1 overexpression had no effect on NRF2 mRNA but decreased NRF2 protein expression (Figures 5(c) and 5(d)), suggesting that Nrf2 expression was regulated by Smurf1-mediated degradation.

**Figure 6:**
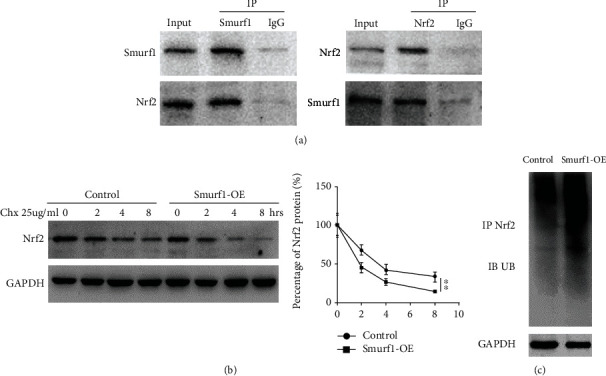
Smurf1 promotes the ubiquitination and degradation of Nrf2. (a) Smurf1 directly interacts with Nrf2 was assessed by CO-IP. (b, c) Western blot analysis of Nrf2 protein stability in Smurf1-OE LF cells treated with 25 *u*g/ml CHX for various times. ^∗∗^*p* < 0.01. (d) Cell lysates from control and Smurf1-OE LF cells was immunoprecipitated with anti-Nrf2 antibody, then assesed by western blot using antiubiquitin antibody.

A reciprocal co-immunoprecipitation assay was performed to further confirm Nrf2 status as the substrate of Smurf1. As shown in Figure 6(a), positive Nrf2 signal was detected in the protein complex pulled down by the anti-Smurf1 antibody. Similarly, a positive Smurf1 signal was also detected in the protein-complex pulled down by the anti-Nrf2 antibody, suggesting that Nrf2 is the direct target of Smurf1. Furthermore, the protein stability of Nrf2 was verified by the cycloheximide assay (CHX) in LF cells with or without Smurf1 overexpression.  Figure 6(b) showed that Nrf2 protein stability was markedly decreased in the Smurf1 overexpression cell. Next, the ubiquitination of Nrf2 was analyzed by CO-IP with an anti-Nrf2 antibody and subsequent immunoblotting with an anti-ubiquitin antibody.  Figure 6(c) showed that Smurf1-OE significantly increased the ubiquitination of Nrf2 in LF cells. Taken together, our data suggest that Smurf1 directly interacts with Nrf2 and accelerates its ubiquitination and degradation.

## 4. Discussion

Lumbar spinal stenosis (LSS) is one of the most common spinal disorders in aging patients, and is characterized by HLF. Previous studies showed that oxidative stress and fibrosis contribute to the progression of HLF [4]. However, the underlying mechanism is unclear. In the current study, we demonstrated that Smurf1 facilitates oxidative stress and fibrosis of ligamentum flavum by promoting Nrf2 ubiquitination and degradation, as evidenced by: (1) fibrosis and oxidative stress were upregulated in LF tissues from HLF patients; (2) Smurf1 was upregulated in LF tissues from HLF patients; (3) Smurf1 facilitated the fibrosis and oxidative stress of LF cells; (4) Smurf1 facilitated the fibrosis and oxidative stress of LF cells by facilitating the ubiquitination and degradation of Nrf2. Fibrosis have been identified as a key process during the development of HLF [4]. As is known, oxidative stress is an important factor in aging-related diseases and usually contributes to the pathogenesis of many diseases by regulating tissue fibrosis [10]. Mohammed et al. reported that necroptosis-mediated inflammation contributes to the fibrosis of the liver and accelerated aging in a mouse model of increased oxidative stress [26]. Hecker et al. demonstrated that loss of cellular redox homeostasis accelerates profibrotic myofibroblast phenotypes that lead to persistent fibrosis associated with aging [27]. Wang et al. demonstrated that in response to oxidative stress, FBW7 regulates cell senescence and tissue fibrosis through telomere uncapping [13]. Similarly, Chuang et al. found that oxidative stress mediates age-related HLF by promoting fibrosis via activating MAPK and AKT pathways [10]. Consistent with previous studies, we also found that oxidative stress and fibrosis was markedly increased in HLF patients compared with LDH control. However, the regulatory mechanism of oxidative stress and fibrosis dysregulation in HLF is still unclear.

Previous studies suggest that protein ubiquitination is an important regulatory posttranslational modification controlling oxidative stress and fibrosis [28]. In the current study, we provide mechanistic insight that Smurf1 is involved in the development of HLF by regulating oxidative stress and fibrosis through Nrf2 ubiquitination. First, our data showed that Smurf1 was upregulated in HLF patients, and overexpression of Smurf1 promoted the oxidative stress and fibrosis of ligamentum flavum cells. Consistent with our findings, other studies have also clarified that Smurf1 promotes the development of multiple fibrosis-related diseases. Qi et al. reported that miR-129-5p targeted Smurf1 and repressed the ubiquitination of PTEN, thus improving the fibrosis and oxidative stress of cardiac in CHF rats [29]. Chen et al. demonstrated that connexin32 ameliorated kindey fibrosis in diabetic mice by accelerating polyubiquitination and degradation of Nox4 by inhibiting Smurf1 expression [30]. Second, our data showed that Nrf2 is the target of Smurf1, and Smurf1 promoting Nrf2 ubiquitination and degradation. Growing studies indicate that Nrf2 is an important negative regulator of oxidative stress and fibrosis. Mohs et al. demonstrated that the activation of Nrf2 in patients with NASH correlates with the grade of inflammation, and in vivo data suggested that NRF2 activation in chronic liver disease is protective by alleviating fibrogenesis and progression of HCC [31]. Marrone et al. reported that KLF2 upregulation profoundly alleviated fibrosis and oxidative stress of HSC partly via the activation of Nrf2 [32]. In addition, ubiquitination and proteasome-mediated degradation of Nrf2 have been documented. Liu et al. reported that BDH2 accelerated the ubiquitination and degradation of Nrf2 and increased the accumulation of ROS [33]. Chen et al. reported that IKK facilitates the ubiquitination of Nrf2 and further promotes oxidative stress-mediated injury of the kidney in obesity-related nephropathy [34]. Here, we reveal that Smurf1 promotes the ubiquitination and degradation of Nrf2, thus, promoting oxidative stress and fibrosis of LF.

## 5. Conclusions

In the current study, we clarify the regulatory mechanism of LF fibrosis and oxidative stress and uncovers a specific E3 ubiquitin ligase, Smurf1, in the development of HLF.

## Figures and Tables

**Table 1:** Patient demographics.

	LDH	HLF	*p* value
Number of cases	12	15	
Sex (female/male)	3/9	7/8	0.247
Age (years)	35.00 ± 10.18	69.27 ± 4.65	< 0.001
LF thickness (mm)	2.62 ± 0.56	5.37 ± 0.73	< 0.001

LDH: lumbar disc herniation; HLF: hypertrophy of the ligamentum flavum.

## Data Availability

The raw data used to support the study's findings is given in the article's figures.

## Supplementary Materials

Supplementary Materials Nrf2 (gene name: NFE2L2) was found to be a potential target of Smurf1 using UbiBrowser.
