# Supplementary figures and images for: Smurf1 Facilitates Oxidative Stress and Fibrosis of Ligamentum Flavum by Promoting Nrf2 Ubiquitination and Degradation
Source: Mediators Inflamm. 2023 Apr 8;2023:1164147. doi: 10.1155/2023/1164147 (PMC10118886; doi:10.1155/2023/1164147)

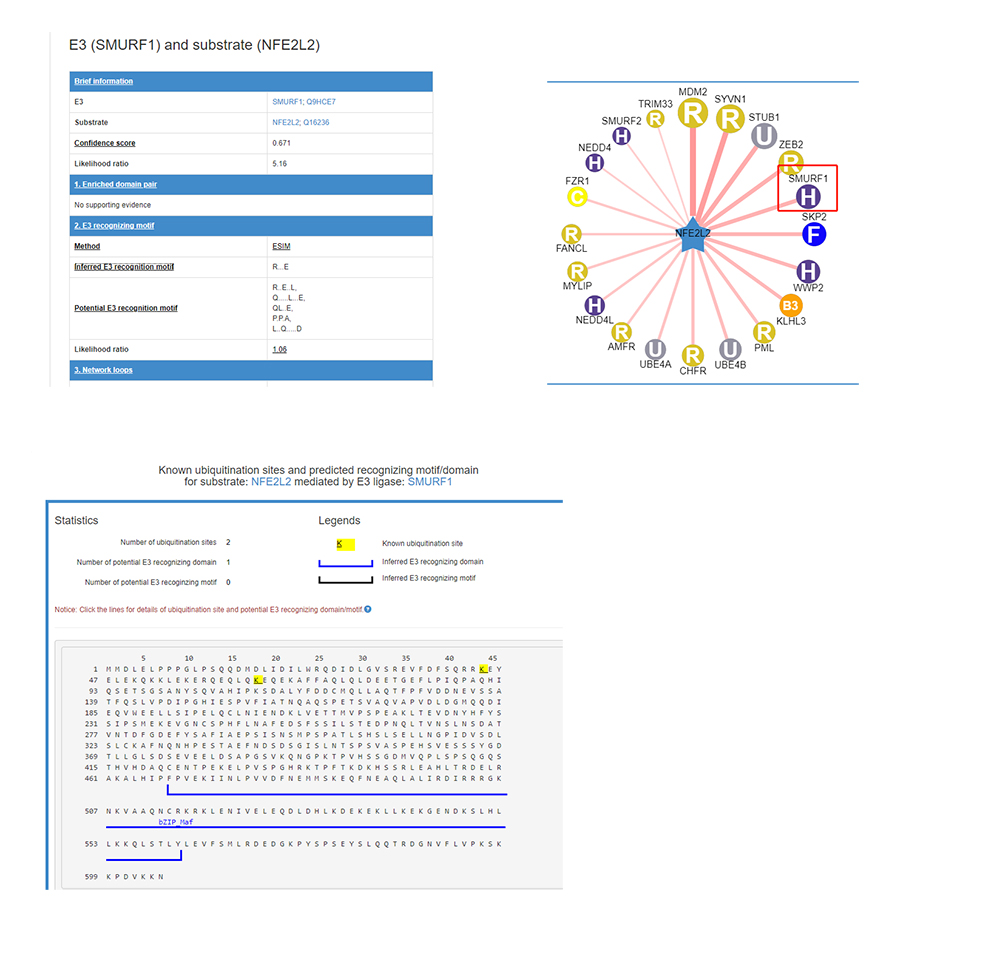

Supplement: Supplementary Materials — Nrf2 (gene name: NFE2L2) was found to be a potential target of Smurf1 using UbiBrowser. [file 1164147.f1.jpg]
